# Labour market participation and sick leave among patients diagnosed with myasthenia gravis in Denmark 1997–2011: a Danish nationwide cohort study

**DOI:** 10.1186/s12883-016-0757-2

**Published:** 2016-11-17

**Authors:** Asger Frost, Marie Louise Svendsen, Jes Rahbek, Christina Malmose Stapelfeldt, Claus Vinther Nielsen, Thomas Lund

**Affiliations:** 1 The National Rehabilitation Centre for Neuromuscular Disorders, Kongsvang Allé 23, DK-8000 Aarhus, Denmark; 2 DEFACTUM, Olof Palmes Allé 15, DK-8200 Aarhus N, Denmark; 3 DEFACTUM, P.P. Oerums Gade 11, Building 1B, DK-8000 Aarhus C, Denmark; 4 DEFACTUM and Section of Clinical Social Medicine and Rehabilitation, Department of Public Health, Aarhus University, P.P. Oerums Gade 11, Building 1B, DK-8000 Aarhus C, Denmark; 5 Danish Ramazzini Centre, Department of Occupational Medicine, University Research Clinic, Regional Hospital West Jutland, Gl. Landevej 61, DK-7400 Herning, Denmark

**Keywords:** Myasthenia gravis, Labour market participation, Sick leave, Cohort study

## Abstract

**Background:**

To examine labour market participation and long-term sick leave following a diagnosis with myasthenia gravis (MG) compared with the general Danish population and for specific subgroups of MG patients.

**Methods:**

A nationwide matched cohort study from 1997 to 2011 using data from population-based medical and social registries. The study includes 330 MG patients aged 18 to 65 years old identified from hospital diagnoses and dispensed prescriptions, and twenty references from the Danish population matching each MG patient on age, gender, and profession. Main outcome measures are labour market participation (yes/no) and long-term sick leave ≥9 weeks (yes/no) with follow-up at 1- and 2 years after the time of MG diagnosis or match. Based on complete person-level information on all public transfer payments in Denmark, persons having no labour market participation are defined as individuals receiving social benefits for severely reduced workability, flexijob, and disability pension.

**Results:**

MG is consistently associated with higher odds of having no labour market participation and long-term sick leave compared with the general Danish population (no labour market participation & ≥9 weeks sick leave at 2-year follow-up, adjusted OR (95% CI): 5.76 (4.13 to 8.04) & 8.60 (6.60 to 11.23)). Among MG patients, females and patients treated with both acetylcholinesterase inhibitors and immunosuppression have higher odds of lost labour market participation and long-term sick leave.

**Conclusions:**

This study suggests that MG patients have almost 6 times higher odds of no labour market participation and almost 9 times higher odds of long-term sick leave 2 years after diagnosis compared with the general Danish population. In particular female MG patients and patients treated with both acetylcholinesterase and immunosuppression have high odds of a negative labour market outcome. Future research should focus on predictors in workplace and labour market policy of labour market participation among MG patients.

## Background

Myasthenia gravis (MG) is a chronic, autoimmune, neuromuscular disease with a prevalence of approximately 20 per 100,000 [1]. The annual incidence is reported as being 1.7–10.4 patients per 1,000,000 residents [1]. Incidence rates have increased in recent years, mostly due to improvement in diagnostics. Advances in medical therapy have continuously increased the life expectancy of MG patients without definitively curing the disease [1–4]. MG affects mainly females (about 70%) and two age-related incidence peaks can be detected among females and men: among females between 20 and 40 years and among men between 60 and 80 years of age [1, 5, 6]. Females constitute up to 70% of all MG patients below 40 years of age [6].

The majority of scientific literature on MG focuses on epidemiology, immunopathogenesis, clinical presentations, diagnosis, and treatment of MG, including emerging therapeutic strategies [1, 7–10]. As life expectancy for MG patients has increased due to advances in treatment and a general increase in life expectancy, research in the past decade has increasingly focused on the consequences of MG in terms of quality of life. These studies indicate a reduction in health-related quality of life (HRQoL), compared to normative values or control groups, and this negative effect is more prominent in physical domains [11–14].

However, only little attention has been devoted to the social consequences of MG. Studies have shown that only 30% of a MG population was employed [15], and that MG is associated with increased risk of long periods of unemployment [16]. It has also been shown, that employment is associated with improved quality of life among MG patients [15]. Therefore, the aim of this study was to analyze the labour market trajectories in terms of labour market participation and long-term sick leave following a MG diagnosis compared with the general population, and for specific groups of MG patients.

## Methods

This nationwide matched cohort study was conducted in Denmark from 1997 to 2011 based on prospectively collected data from population-based medical and social registries. Since 1968, all Danish residents have been assigned a unique 10-digit personal identification number, which is used in all databases and allows for explicit person-level linkage between databases [17]. The study was approved by the Danish Data Protection Agency (journal no. 2012-41-0867).

### Data sources

The Danish National Patient Register holds records of all patients admitted to Danish somatic hospitals since 1977 and outpatient activities, emergency room contacts, and activities in psychiatric wards since 1995. The Registry contains administrative and clinical data for both inpatient- and outpatient hospital contacts, including discharge diagnoses coded according to the International Classification of Diseases (ICD), version 8 until 1994 and subsequently version 10 [18].

The Danish National Prescription Registry includes data on all prescriptions dispensed at outpatient pharmacies since 1994. The registry contains information on the drug user, the dispensing, the prescriber and the pharmacy, including information on the date of each dispensing and the global Anatomical Therapeutic Chemical classification (ATC) code identifying each dispensed drug in a five-level classification system [19].

The Danish Register for Evaluation of Marginalisation (DREAM) holds person-level information on all public transfer payments since 1991 registered on a weekly basis. The register contains more than 100 specific codes for transfer income, including detailed coding on e.g. unemployment, leave of absence, retirement, sick leave, social assistance (transfer income administered by the municipal social service department), grants from the State Education Fond, and flexijob (job created for persons with limited work capacity applying normal wage and a public benefit transferred to the employer) [20].

Other nationwide population-based Danish registries give access to person-level sociodemographic characteristics on e.g. profession, marital status, and vital status [21, 22].

### MG patients

Patients with MG were included if 1) aged 18–65 years old and 2) registered in The Danish National Patient Register with MG (ICD-10 DG70.0) including both inpatient and outpatient hospital contacts, and 3) having ≥2 dispensed prescriptions with acetylcholinesterase inhibitors (AChEI) (ATC-code N07AA02) [23]. A total of 399 MG patients fulfilled these criteria during 1 January 1997 to 31 December 2011. A nationwide clinical database for MG neurorehabilitation further identified 6 MG patients during 1997–2011 of whom 4 patients had hospital contact for MG but did not have ≥2 dispensed AChEI prescriptions, and 2 MG patients only had ≥2 dispensed AChEI prescriptions. Among these 405 MG patients, we excluded 75 MG patients who had no labour market participation, were retired, or were unavailable for follow-up at baseline assessed 1 year before diagnosis due to a varying time span from symptom onset until being diagnosed, leaving a total of 330 MG patients.

### Study population

The reference group consisted of randomly selected subjects without MG from the Danish population. Only references having no hospital contact in the year of MG diagnosis were included. Twenty references were selected to match each of the 330 MG patients at the time of diagnosis on integer values of age, gender, and approximately 100 categories of profession prior to diagnosis/match. The registry establish profession ultimo November each year for all Danish citizens.

### Outcome variables

Follow-up on labour market participation and long-term sick leave ended after 1 and 2 years from the date of diagnoses for MG patients and date of matching for references. Labour market participation was based on weekly coding of social transfer payments in DREAM, and dichotomized into “yes” and “no”. The category “yes” includes people who were self-supporting or receiving social benefits for which a minimum of workability is an eligibility criteria, such as sick leave, occupational rehabilitation, unemployment, education, and maternity leave. The category “no” includes people receiving social benefits granted for severe reduced workability, flexijob or unemployed as such, and disability pension. Research indicates that sick leave length, and in particular more than 8 weeks of absence, is a predictor of future reduced workability [24], and weeks of sick leave from the time of diagnosis/match were therefore assessed in categories of 0–8 weeks and 9–52 (1-year follow-up) and 0–8 weeks and 9–104 (2-years follow-up), respectively.

### Co-variables

Baseline data at the time of diagnosis were included on age, gender, marital status, profession, weeks of sick leave 1 year prior to diagnosis/match, and time period. Pharmacological treatment with both acetylcholinesterase inhibitors (ATC-code N07AA02) and immunosuppression was also included as a covariate since initial treatment with acetylcholinesterase inhibitors is generally supplemented with immunosuppression when MG symptoms are not adequately controlled, and MG treatability may likely associate with prognosis [1]. Treatment with immunosuppression includes the following ATC-codes: S01BA04 (prednisolone), L04AX01 (azathioprine), L04AA06 (mycophenolic acid), L04AD01 (ciclosporin), L04AD02 (tacrolimus), L01AA01 (cyclophosphamide), or L01BA01 (methotrexate).

### Statistical analysis

Age was included as an integer variable, but was categorized into early onset (18–39 years) versus late onset (40+ years) [5, 6] when analyzing the association between age and labour market outcome among MG patients. Baseline data on profession was classified in 4 groups (employer/director, employee, not employed, pension/early retirement). Although MG patients on retirement 1 year before diagnosis were excluded from analyses, some patients changed labour market status before November when profession was assessed, and as a result the baseline data on profession includes a category for persons on pension/early retirement. Due to a non-normal distribution, weeks of prior sick leave were categorized into 0 weeks (no sick leave), 1–8 weeks (short-term sick leave) and 9+ weeks (long-term sick leave). Time period was categorized into 1/1 1997-1/6 2008 and 2/6 2008-1/1 2012 reflecting that Danish employers received no reimbursement of sickness benefit from the state in the first 14/15 days of sick leave during the period 1/1 1997 to 1/6 2008 and in the first 21 days during the period 2/6 2008 to 1/1 2012.

Baseline characteristics of MG patients and matched references were compared with means, standard deviations and percentages. The associations between MG and labour market participation as well as sick leave were assessed 1 and 2 years after diagnosis/match using logistic regression adjusting for the co-variables and data categories shown in Table 1 to obtain odds ratios (OR) and 95% confidence intervals (CI). Similar analyses were performed exclusively in MG patients to evaluate the association between specific patient characteristics and labour market participation as well as sick leave. Less than 0.1% of MG patients and matched references had missing data on marital status (Table 1), and these patients were excluded from the adjusted analyses.

**Table 1:** Baseline characteristics: a national cohort of patients with myasthenia gravis vs. matched references

	Myasthenia gravis, *n* = 330	Matched references; *n* = 6600
Characteristics		
Age, m (sd)	42.7 (13.0)	42.7 (13.0)
Male, *n* (%)	130 (39.4)	2600 (39.4)
Profession, *n* (%)		
Employer/director	31 (9.4)	620 (9.4)
Employee	240 (72.7)	4800 (72.7)
Not employed	57 (17.3)	1140 (17.3)
Pension/early retirement	2 (0.6)	40 (0.6)
Marital status, *n* (%)		
Married/registered partnership	197 (59.7)	3712 (56.2)
Not married/registered partnership	130 (39.4)	2885 (43.7)
Missing	3 (0.9)	3 (<0.1)
Pharmacological treatment, *n* (%)		
AChEI	158 (47.9)	–
AChEI+	172 (52.1)	–
Prior sick leave (1 year), *n* (%)		
0 weeks	190 (57.6)	5851 (88.7)
1–8 weeks	80 (24.2)	477 (7.2)
9–52 weeks	60 (18.2)	272 (4.1)
Time period		
1/1 1997–1/6 2008	249 (75.5)	4980 (75.5)
2/6 2008–31/12 2011	81 (24.6)	1620 (24.6)

*Abbreviations*: *AChEI* Acetylcholinesterase inhibitors, *AChEI+* AChEI and immunosuppression

To evaluate the robustness of our findings, the analyses were performed excluding patients who died during follow-up. Furthermore, the analyses were repeated including the 75 MG patients who were excluded from the primary analyses because they had only minimal or no risk of event at baseline or were unavailable for follow-up (and their matched pairs). Finally, the analyses were performed excluding the matched references who had only minimal or no risk of event at baseline or were unavailable for follow-up (and their matched pairs). All sensitivity analyses were performed with logistic regression adjusting for the co-variables in Table 1 to obtain OR and 95% CI.

All analyses were performed in Stata 13 as two-tailed analyses considering *p* < 0.05 statistically significant.

## Results

A total of 2 MG patients (0.6%) and 4 matched references (0.1%) died during 1-year follow-up, and 3 MG patients (0.9%) and 21 matched references (0.3%) died during 2-year follow-up. MG patients and the matched references were compatible with regard to age, gender, and profession with a mean age of 42.7 years, females constituting 60.6% of both groups, and more than 80% were in employment. Slightly more of the MG patients were married or in registered partnership (60.2%) than among the matched references (56.3%). Among the MG patients, 47.9% received pharmacological treatment with solely acetylcholinesterase inhibitors, whereas the remaining 52.1% received further treatment with immunosuppression. MG patients more often had a labour market history involving sickness absence 1 year before diagnosis compared to the matched references (Table 1).

Initial analysis of labour market participation and sick leave after time of diagnosis showed that MG patients subsequently experienced labour market trajectories with generally poorer labour market participation than the matched references both at 1- and 2-year follow-up, and more often suffered from long-term sick leave. In the first year after diagnosis, 41.2% of the MG patients experienced 9 or more weeks of sickness absence. The corresponding figure in the reference group was 3.4%. This trend was also observed at 2-year follow-up, where 47% of MG patients had been sick-listed for more than 9 weeks. The corresponding figure among the matched references was 6.8% (Table 3).

Logistic regression analysis showed that MG patients had significantly higher odds of having no labour market participation. The odds were more than two-fold after 1 year, and almost a factor 6 at the 2-year follow-up. The association between pharmacological treatment and labour market participation was only significant after 2 years, where those receiving both acetylcholinesterase inhibitors and immunosuppression had an OR of 1.95 for no labour market participation compared to those receiving solely acetylcholinesterase inhibitors. There were no differences in labour market participation according to age among MG patients. Female MG patients had significantly higher odds of lost labour market participation at 2 years, compared to male MG patients (Table 2).

**Table 2:** Association between myasthenia gravis and labour market participation during 1- and 2-years follow up

	No LMP, *n* (%)	Unadjusted OR (95% CI)	Adjusted OR (95% CI)^a^
Comparison groups	No LMP, 1 year after diagnosis/match		
MG vs. matched references	35 (10.6)/309 (4.7)	2.42 (1.67–3.49)	2.23 (1.46–3.38)
MG: age 40+ vs. 18–39	22 (11.2)/13 (9.7)	1.18 (0.57–2.43)	2.02 (0.84–4.86)
MG: male vs. female	10 (7.7)/25 (12.5)	0.58 (0.27–1.26)	0.67 (0.28–1.59)
MG: AChEI+ vs. AChEI	21 (12.2)/14 (8.9)	1.43 (0.70–2.92)	1.26 (0.57–2.78)
Comparison groups	No LMP, 2 years after diagnosis/match		
MG vs. matched references	75 (22.7)/327 (5.0)	5.64 (4.26–7.47)	5.76 (4.13–8.04)
MG: age 40+ vs. 18–39	44 (22.5)/31 (23.1)	0.96 (0.57–1.62)	1.21 (0.64–2.27)
MG: male vs. female	19 (14.6)/56 (28.0)	0.44 (0.25–0.78)	0.39 (0.20–0.74)
MG: AChEI+ vs. AChEI	49 (28.5)/26 (16.5)	2.02 (1.18–3.45)	1.95 (1.09–3.50)

*Abbreviations*: *LMP* labour market participation, *MG* myasthenia gravis, *AChEI* Acetylcholinesterase inhibitors, *AChEI+* AChEI + immunosuppression. *OR* odds ratio, *CI* confidence interval

^a^Adjusted for age, gender, profession, marital status, pharmacological treatment, sick leave 1 year before diagnosis/match, and time period

MG patients had profound excess odds of sickness absence exceeding 9 weeks at both 1 and 2-year follow-up compared with matched references. The OR for MG patients at 1 and 2 years were 14.8 and 8.6 respectively in the adjusted models. Among MG patients, age, gender and pharmacological treatment were not associated with sick leave at 1-year follow-up. At 2-year follow-up, most adjusted models showed excess odds of sickness absence among females, whereas age and pharmacological treatment were not significantly associated with sick leave (Table 3).

**Table 3:** Association between myasthenia gravis and sick leave during 1 and 2-years follow up

	≥9 weeks, *n* (%)	Unadjusted OR (95% CI)	Adjusted OR (95% CI)^a^
Comparison groups	≥9 weeks sick leave, 1 year after diagnosis/match		
MG vs. matched references	136 (41.2)/225 (3.4)	19.86 (15.37–25.67)	14.80 (10.97–19.95)
MG: age 40+ vs. 18–39	84 (42.9)/52 (38.8)	1.18 (0.76–1.85)	1.25 (0.70–2.24)
MG: male vs. female	50 (38.5)/86 (43.0)	0.83 (0.53–1.30)	0.66 (0.38–1.15)
MG: AChEI+ vs. AChEI	76 (44.2)/60 (38.0)	1.29 (0.83–2.01)	1.17 (0.70–1.96)
Comparison groups	≥9 weeks sick leave, 2 years after diagnosis/match		
MG vs. matched references	155 (47.0)/448 (6.8)	12.16 (9.60–15.41)	8.60 (6.60–11.23)
MG: age 40+ vs. 18–39	94 (48.0)/61 (45.5)	1.10 (0.71–1.71)	1.34 (0.76–2.36)
MG: male vs. female	53 (40.8)/102 (51.0)	0.66 (0.42–1.0)	0.50 (0.29–0.86)
MG: AChEI+ vs. AChEI	91 (52.9)/64 (40.5)	1.65 (1.07–2.55)	1.61 (0.98–2.64)

*Abbreviations*: *MG* myasthenia gravis, *AChEI* Acetylcholinesterase inhibitors, *AChEI+* AChEI + immunosuppression, *OR* odds ratio, *CI* confidence interval

^a^Adjusted for age, gender, profession, marital status, pharmacological treatment, sick leave 1 year before diagnosis/match, and time period

The results in Tables 2 and 3 were largely confirmed by analyses including only survivors. In the two analyses either including MG-patients or excluding matched references with minimal or no risk of event at baseline (and their matched pairs), all point estimates suggested that MG patients above 39 years have higher odds of having no labour market participation than MG patients younger than 40 years, although not all results reached statistical significance.

## Discussion

The study showed that MG patients more often had a labour market history involving sickness absence than the matched references, and generally experienced poorer labour market participation and suffered from long-term sick leave at 1- and 2 year follow-up, respectively. The excess odds of having no labour market participation among MG patients were approximately 2- and 6-fold at 1- and 2-year follow-up. The ORs among MG patients for sickness absence exceeding 9 weeks at the two follow-ups were approximately 15 and 9, respectively. With regard to gender, female MG patients had significantly higher odds of experiencing long-term sickness absence and having no labour market participation at 2 years after time of diagnosis, than male MG patients. The results were largely confirmed by several sensitivity analyses.

### Strengths and weaknesses of the study

A main strength of this study is the prospective study design, the large nationwide cohort identified from both inpatient and outpatient hospital contacts as well as dispensed prescriptions, and the detailed and complete data on labour market participation [20]. All data were prospectively collected unaware of the study hypotheses, minimizing the risk of selection and information bias. Furthermore, confounding was minimized by matching and adjusting for potentially strong confounders, including previous sickness absence [25], profession [26, 27], marital status [27], and gender [26, 27]. Although residual confounding may exist, adjustment for these factors represents a considerable strength of the study.

Because of the observational design, the results may be affected by unaccounted confounding from e.g. work environment and health behaviour, modifying the observed associations in an unknown direction [27, 28]. Co-morbid diseases may also be a potential confounder [26, 29]. However, research shows that life expectancy in MG patients do not differ significantly from the general population which was also confirmed in our study where only minor differences in mortality were seen between MG patients and the general population [29]. This may indicate that MG patients and non-MG patients were balanced with respect to prognostic factors with heavy impact on mortality, including a range of co-morbid diseases. The observed minor differences in mortality may be a result of matching where only individuals with no hospital contacts in the year of MG diagnosis were selected which may potentially introduce bias towards stronger associations between MG and poor labour market participation. Furthermore, misclassification in labour market outcomes may exist due to a flexible Danish labour market enabling different possible working scenarios for MG patients, e.g. MG patients working part-time without transfer payment instead of having transfer payments due to part-time sick leave. The direction and magnitude of this potential bias is unknown. However, the observed findings are despite these fallacies substantiated by the pronounced strength of associations, the plausible findings and consistency in results compared with other research [30].

### Comparison with other studies

Research from other countries supports our findings that MG is associated with increased sickness absence and loss of labour market participation. An Australian study showed that approximately 40% out of 165 MG patients stopped working due to MG, and nearly 20% changed occupation or reduced work hours (mean disease duration not published) [31]. During a 1-year period, about one fifth of the patients were on sick leave of which 7% were on long-term sick leave (≥9 weeks) [31]. A German study including 1518 MG patients with mean disease duration of 10.2 years found that only 25.5% of the patients worked more than 15 h per week, and almost 70% had no labour market participation [15]. An Italian study of 101 MG patients showed that 20% of the patients stopped working prior to retirement age [32]. The studies are hardly comparable with this study because of differences in study design, outcome measures, and follow-up periods, but all studies suggest that MG is negatively correlated with labour market participation and sickness absence. We are unaware of any studies assessing the causal mechanisms in the link between MG and weak labour market participation. However, a study has shown that both a Dutch and Norwegian MG population had reduced HRQol assessed by SF-36, primarily caused by limitations in their physical health leading to restrictions in participation in everyday life [33]. Generalized fatigue in addition to muscle fatigue is a frequent symptom and has reached a growing focus [34]. Furthermore, concomitant autoimmune diseases and side-effects by treatment may also have a negative influence on labour market participation [29, 30]. Our study suggests that MG have a particularly high negative impact on sick leave during the first year after diagnosis, and a high negative impact on labour market participation during the second year after diagnosis compared with the general Danish population. This may indicate that a disproportion exists between patient resources and the workplace- or labour market requirements and adaptations which may in the long term hinder MG patients from participating in the workforce [30]. Future research should focus on predictors in workplace and labour market policy of labour marked participation among MG patients in order to optimize efforts to keep the MG patients in the workforce.

MG is characterized by fluctuating symptoms but studies suggest that MG symptoms and optimized treatment have stabilized for up to 90% of the patients by 3 years after MG onset [35, 36]. However, the exact time of disease onset is difficult to assess and the diagnostic period most likely varies across countries, probably depending on the clinical presentation of the patients, including the AchR-serologic profile, and differences in health care organization. Likewise differences exist in labour market policies between countries with a high impact on labour market participation. Although this study includes a complete national population of MG patients over 18 years, special attention must be given to specific health care organization and labour market policies before generalizing the results to other countries and settings.

## Conclusions

The study shows that MG is associated with higher odds of having no labour market participation and increased long-term sick leave compared with the general Danish population during the first 2 years after diagnosis. In particular female patients and patients treated with both acetylcholinesterase inhibitors and immunosuppression have higher odds of lost labour market participation and long-term sick leave than male patients and patients treated with acetylcholinesterase inhibitors alone. Future research should identify predictors in workplace and labour market policy of labour marked participation among MG patients.

## Abbreviations

AChEI: Acetylcholinesterase inhibitors
ATC: The Global Anatomical Therapeutic Chemical classification
CI: Confidence intervals
DREAM: The Danish Register for Evaluation of Marginalisation
HRQoL: Health-related quality of life
ICD: International Classification of Diseases
MG: Myasthenia gravis
OR: Odds ratios
